# Prevalence and factors influencing substance abuse among secondary school students in Mbeya City, Tanzania

**DOI:** 10.4314/ahs.v24i3.47

**Published:** 2024-09

**Authors:** Abnery Gift, Clement N Mweya

**Affiliations:** 1 Mbeya College of Health and Allied Sciences, University of Dar es Salaam, P.O.Box 608, Mbeya, Tanzania; 2 Mbeya Medical Research Centre, National Institute for Medical Research, P.O.Box 2410, Mbeya, Tanzania

**Keywords:** Substance abuse, cannabis, alcohol, secondary school students, Tanzania

## Abstract

**Background:**

Substance abuse among students has far-reaching social, religious, economic, health and demographic consequences, such as unplanned pregnancies, school dropouts, abortions, maternal and newborn mortality, infection risks and other psychological problems. Our study investigated the prevalence of substance abuse among secondary school students in Mbeya City, Tanzania.

**Methods:**

This school-based cross-sectional study was conducted from January to May 2021 among secondary students from Mbeya City, Tanzania. Students completed a self-administered substance use questionnaire. Data were analysed using descriptive statistics.

**Results:**

A total of 343 students were requested to participate in the study. However, 300 agreed to indicate 87% response rate. 9.7% (n=59) reported substance use, primarily cannabis, 75% (n=225) and alcohol, 68% (n=204). Friends influenced substance use among 82.7% of users. Reported immediate effects included misbehaviour, 73.7% (n=221) and unprotected sex, 72.3% (n=217). Long-term harms were predominantly brain damage (86.7%). Community education (78%) and media campaigns (76.7%) were suggested to curb use.

**Conclusion:**

Substance abuse among a sizable minority of Mbeya City secondary school students is driven by peer influence and has detrimental impacts reported by students themselves. Comprehensive education and preventive strategies engaging families, schools and communities are recommended to address this critical youth issue.

## Introduction

Substance abuse is a non-adaptive model of substance use that leads to a slew of issues and negative consequences.^1^ It manifests itself in cognitive, behavioural and psychological ways.^2^ The American Psychiatric Association defines substance abuse as using drugs or alcohol that results in legal issues, causes distress or difficulties functioning in significant areas of life, or happens in risky settings.^3^ Many youths worldwide are prone to substance abuse, as documented in various studies.^1^,^4^,^5^ Substance abuse among youth has far-reaching social, religious, economic, health and demographic consequences. Youth is a critical period in which values are formed.^6^ Substance dependence during youth results in chronic abuse characterised by symptoms such as tolerance and withdrawal. Tolerance is a condition resulting from repeated use that typically occurs with alcohol, barbiturates, amphetamines, and opiates such as morphine and heroin. Withdrawal is a group of physical and psychological symptoms when a drug is withheld after the body has adjusted to its repeated administration.^3^ Prolonged use is associated with undesirable outcomes such as unplanned pregnancies, school dropouts, abortions, maternal and newborn mortality, infection risk, HIV/AIDS infection, brain problems and other related impacts.^7^

Factors leading to the initiation of substance abuse have been identified, and many intervention strategies have been established, including drug abuse prevention programmes that include demand reduction, drug prevention in school, management of free time, and government responsibility toward eradicating drugs. Many young individuals begin to experiment with various things in adolescence, including social partnerships and the major issue of substance abuse. In studies conducted in Nigeria and Ethiopia, results showed that 33.7% and 45.5% of the respondents were substance abusers, respectively,^8^,^9^ suggesting that the prevalence of substance use has an increasing trend. Similarly, regarding adolescence and drug abuse in northern Tanzania, the study findings revealed that seven per cent of secondary school students experienced drug use such as alcohol, cigarettes, cannabis and khat at a young age.^10^ In rural America, young adults living in rural-large areas have higher rates of substance abuse than their urban peers; those living in the most rural areas had nearly twice the rate of methamphetamine use as urban young adults. Rural youth are more likely than urban youth to engage in the high-risk behaviour of driving under the influence of alcohol or other illicit drugs.^11^

Students need to understand the extent of drug utilization among adolescents because of their age. They have an opportunity to have access to education compared to those who are not in school in matters concerning the potential lifetime effects of substance abuse. There are many reasons adolescents use these substances, including the desire for new experiences, simple peer pressure alleviation of stress, social acceptance, lower parents' education level, and the desire to attain a high personality profile. Adolescents are “biologically wired” to seek new experiences, take risks, and carve out their identities.^9^ According to studies done worldwide, there is a big gap between prevalence among other countries and Tanzania. Our study aims to lessen that gap by elucidating awareness of the impacts and factors contributing to substance abuse among secondary students so that schools can implement targeted government prevention programmes.

## Methods

### Study area

This study was conducted in Mbeya City, southwestern Tanzania, near the borders with Zambia and Malawi. Its reported coordinates are approximately 8°54′ South and 33°27′ East. As of 2019, Tanzania had over 5,100 public and private secondary schools. Mbeya region has a total of 247 Ordinary level secondary schools (173 government and 74 private) and 55 Advanced secondary education schools (23 government and 32 private).^12^ Mbeya City has 75 Secondary Schools, of which 55 are Ordinary level Secondary Schools (the government owns 31 and 24 are private) and 20 are Advanced level Secondary Schools (the government owns 4 and 16 are private).^13^ In 2019, over two million students enrolled in secondary education in Tanzania. Female students were higher in number compared to males.^12^ Mbeya City was selected as the study area due to its rapidly growing population and economic development, along with anecdotal reports of increasing substance use among youth in the region. The city has experienced significant population growth and urbanisation in recent decades. The influx of people and businesses has brought changing social dynamics that may influence risk factors for substance abuse among young people. Additionally, the Mbeya region is an important transit hub for trade routes that could enable increased access to illicit drugs. Scientifically, focusing the research in Mbeya City provided an opportunity to gather substantive data on adolescent substance use behaviours in an understudied but potentially high-risk area of the country.

### Study design

This study utilized a school-based cross-sectional design. Data was collected through a survey conducted at selected secondary schools in Mbeya City. The survey was administered during a single time point between January to May 2021. The school-based approach provided direct access to the adolescent study population in their educational setting.

### Study population

All students who were present in school on the day of data collection and who consented to participate were included in the sample, and all students who did not agree to participate were excluded.

### Sampling procedure and variables

A convenient random sampling technique was used for anyone who happened to be at school on the day of data collection. The dependent variable was the effects of substance abuse, while independent variables included factors influencing use, such as peer pressure and stress relief. Demographic factors like age and gender were captured. Students were also asked about sources of information and prevention strategies for substance abuse.

### Sample size estimation

A sample size (n) of 343 participants was calculated using n=Z2P(1-P)/e2 at the margin error (e) of 5% and standard normal deviation of 1.96, corresponding to a 95% confidential interval. The prevalence (P) of 33.7% was used, similar to a study of Psychoactive Substance Abuse amongSecondary School Students in Enugu, Nigeria.^14^

### Data collection

The researchers collected data through a self-administered questionnaire for secondary school students from different schools in Mbeya City. A Swahili version of the questionnaire was used for data collection. A clear definition of substance abuse was provided to students before beginning data collection. Information collected included their prior experience with substance use, types of substance use and factors influencing their use. Students were also asked to express the effects of substance abuse and suggestions on ways to prevent substance abuse among students. Information on questionnaire administrations was shown to study participants, including assurances of confidentiality and use of information obtained for research purposes only.

### Data management and analysis

The collected data was prepared for analysis by checking for any errors or missing values and cleaning the data set.^15^ The questionnaire responses were coded numerically and entered into IBM SPSS Statistics 20.0 software (IBM Corp., Armonk, NY, USA) for statistical analysis. After descriptive statistics, the results were presented in texts and figures.

### Ethical consideration

Approval for conducting this study was obtained from the University of Dar es Salaam, Mbeya College of Health and Allied Science Research Ethical Clearance Sub-Committee. Permission to conduct the study was taken from the school Headmasters or Headmistresses. Informed consent was sought and obtained from all students before agreeing to participate in the study.

## Results

### Prevalence of substance abuse among students

**Figure 1:**
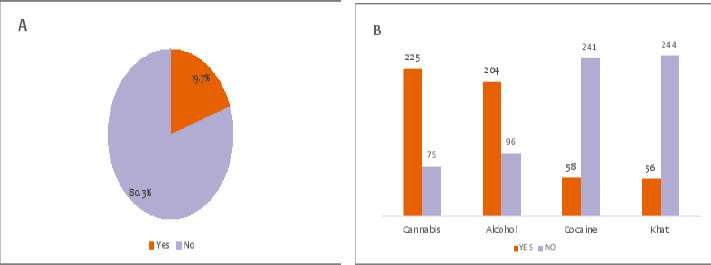
**A** Prevalence of substance abuse among students in Mbeya City, Tanzania **B** Reported substances being abused by students in Mbeya City, Tanzania

A total of 343 students were requested to participate in the study. However, 300 agreed to indicate an 87% response rate. 50.3% were females (n=151) and 49.7% (n=149) were males. The sample included all secondary students aged 13-22 from Mbeya city who participated in this study. A small percentage of students responded about substance abuse (19.7%) (Figure 1A). The most common substance reported being used was cannabis by 75% (n=225), followed by alcohol by 68% (n=204) (Figure 1B).

### Factors influencing substance abuse

**Figure 2:**
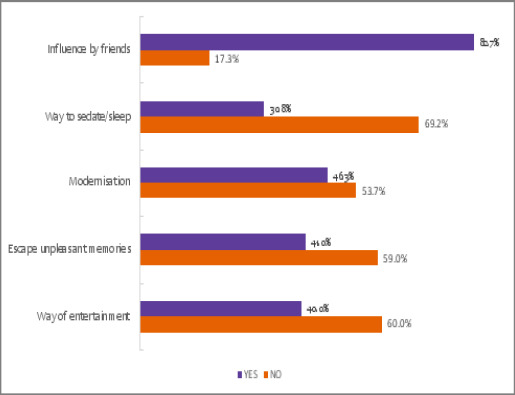
Factors influencing substance abuse in secondary school students in Mbeya City, Tanzania

It was reported that the majority, 82.7% (n=248) of students are influenced by friends to get involved in substance use, whereas other factors such as modernisation, ways to sleep, escape from unpleasant memories and as a way of entertainment were reported as about 40% of students as indicated in Figure 2.

### Reported effects of substance abuse

**Figure 3:**
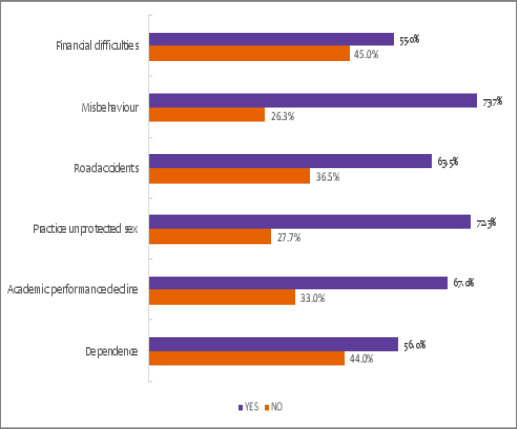
Reported effects of substance abuse among students in Mbeya City, Tanzania

**Figure 4:**
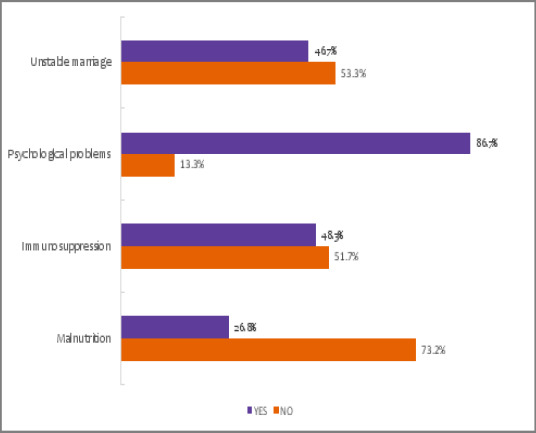
Reported long-term effects of substance abuse among students in Mbeya City, Tanzania

According to the students assessed, the immediate effects of substance abuse are misbehaviours due to central nervous system involvement, as was reported by 73.7% (n=221) students, as well as the practice of unprotected sex, as reported by 72.3% (n=217) of the students (Figure 3). Most students reported that brain damage is the most common long-term effect of administering substances of abuse (Figure 4).

### Reported ways to prevent substance abuse

**Figure 5:**
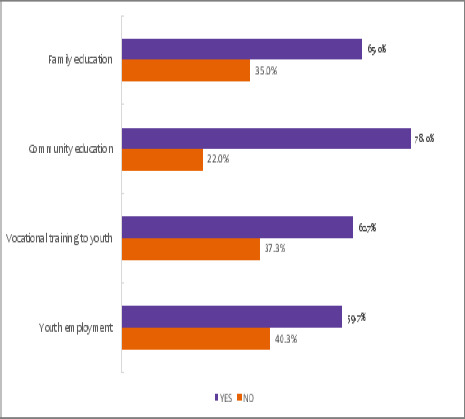
Reported ways for prevention of substance abuse among secondary students in Mbeya City, Tanzania

**Figure 6:**
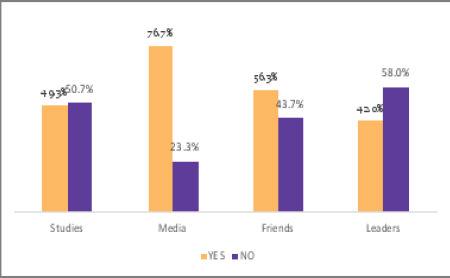
Reported source of information on substance abuse in Mbeya City, Tanzania

78% (n=234) of students suggested that community education by using different ways of communication will play a more significant role in preventing the students from being involved in substance abuse. About 50% suggested other techniques such as family education, vocational training and youth employment as possible ways to prevent substance use among youth (Figure 5). Most students, 76.7% reported the media as their source of information on substance abuse (Figure 6).

## Discussion

Findings from our study indicated that only a few students (19.7%) were found to practice substance abuse. Possibly this is due to fear of respondents giving information about their behaviour on substance abuse. Most of the students who were found to involve themselves in substance abuse had more than one year in school. In similar studies conducted in Nigeria and Ethiopia, results showed that 33.7% and 45.5% of the respondents were substance abusers, respectively.^8^,^9^ Also suggested that the prevalence of cannabis use has an increasing trend. This is supported by the fact that there has been an increase in admissions to the psychiatric department at Muhimbili National Hospital, with complications relating to cannabis and intravenous drug abuse, e.g., heroine.^16^ In a study done in Mwanza, Tanzania, more males (70.7%) than females (29.3%) reported higher substance use.^17^ In adolescence and drug abuse in central and eastern Tanzania, the study findings revealed that 5-15% of young people had experienced drug use such as alcohol, cigarettes, cannabis and khat at a young age, and 2.1% have injected themselves with drugs such as heroin popularly known as “brown sugar”.^18^ In a study done in Iran, almost 6.7% of the participants had a history of drug abuse.^19^

Although Tanzania's laws provide stern punitive measures against all those involved in drug trafficking and consumption, the war against narcotics seems complicated as Tanzania continues to be a transit route for illicit drugs.^18^ A similar study in Nigeria indicated that older students were more involved in numerous cases of abuse than younger ones. The highest prevalence of daily substance use was found among those who used cigarettes and coffee.^8^ Another study in Nigeria reported that the frequency and percentage of responses to items on the questionnaire showed that 33.7% of the respondents were substance abusers. Alcohol was most commonly abused (31.6%), while cannabis was the least (4.1%). The age of initiation of substance use was lower than some reports from other centres. Males consumed most psychoactive substances more frequently than females. Cigarettes and cannabis were the exclusive preserve of the males. Seventy-five per cent of the students were involved in multiple substance abuse.^8^ A study done in Moshi reported that the prevalence of ever use of alcohol among women with partners was 50·8% and slightly higher among women without partners at 57·5%.^20^

Findings from our study indicated that the most abused substance is cannabis, whereby 75% of students reported that cannabis is mostly used in school. These findings are similar to Kalula (2011),^18^, who confirmed that cannabis is a significant problem in Tanzania. Also, the students reported that many students who abuse cannabis with alcohol, whereby a total of 204 (68.0%) reported that alcohol was also used in combination with cannabis. This finding is similar to that by Masibo et al. (2013),^22^ whereby his study showed that many youths reported using cannabis in combination with alcohol. A study in Tanzania showed that youth who are mostly involved in substance abuse are male most of the time, suggesting that substance abuse is a male activity in the Republic of Tanzania.^18^ Another study in Mwanza, Tanzania reported that the most frequently used substances were Alcohol, 59.3%, tobacco, 38.6% and Cannabis, 29.3%, while heroin and cocaine were the least used (2.1% and 1.6%, respectively.^17^ A study in Nigeria indicated alcohol was most commonly abused (31.6%), while cannabis was the least (4.1%).^8^ In a study done in Ethiopia, the most commonly used drugs in descending order were alcohol (25.1%), cigarettes (11.4%), and khat (9.2%). Male participants, urban setting, peer pressure, personal pleasure, academic dissatisfaction and pocket money were highly associated with substance abuse.^9^ In a study done in Mwanza, Tanzania, the most frequently used substances were Alcohol, 59.3%, tobacco, 38.6% and Cannabis, 29.3%, while heroin and cocaine were the least used (2.1% and 1.6%, respectively).^17^ Another study in Nigeria reported that alcohol was most commonly abused (31.6%), while cannabis was the least (4.1%).^8^ In Ethiopia, a study reported that the most commonly used drugs in descending order were alcohol (25.1%), cigarettes (11.4%), and khat (9.2%).^9^

Our findings indicate that factors influencing youth to engage in substance abuse is the influence of their fellow friends (82.7%). Students reported that most students who abuse substances start after being influenced by their friends. It seems that friends have more influence on changing somebody's behaviour despite the fact that students get information on substance abuse mainly from the media, where 230 out of 300 reported using the media as their main source of information. Although students know the potential effects of abuse of substances, the problem is still increasing. Most students reported being aware of the immediate and long-term impact of prolonged administration of substance abuse. A study in Nigeria indicated that the reasons for using substances included relief from stress, 43.5% (n=175), self-medication to treat illness, 23.8% (n=96), and staying awake at night to study, 14.9% (n=60).^14^

Findings from this study showed that most secondary school students (73.7%) suggested that misbehaviour had an immediate effect after substance abuse, according to what they observed from their fellow abusers. Although there are several direct effects, such as poor performance in their studies, dependence, and practising unprotected sex, which may result in STDs and HIV, misbehaviour seems to be well-known in their vicinity. On the side of long-term effects, a total of 260 (86.7%) students suggested that brain damage is a well-known long-term effect simply because it is the CNS system that is highly stimulated. To fight against the abuse of substances, most students (234(78%) suggested that the whole community should have access to education through different ways of communication to make the entire society aware of the risk factors, potential effects, and the way to fight against the abuse of substances. Some students suggested that substance abuse should be part and parcel of their study to make it well understood.

While this exploratory study provides initial evidence of prevalence and risk factors, the limitations constrain the interpretability and application of the results. These limitations restrict the generalizability and validity of the findings. The reliance on a small convenience sample of students from only Mbeya City schools reduces representativeness. Additionally, the self-reported data regarding illegal substance use behaviours may be inaccurate due to underreporting resulting from social desirability biases, poor memory recall and limited student insight into their motivations and experiences. The inclusion of more schools across Tanzania, a larger randomised sample and more objective measures to validate self-reports would improve the research quality.

## Conclusion

Our study shows that only a few students smoke cannabis and drink alcohol due to the influence of friends that drives them to engage in such behaviour. It seems that the root problems lie in their environment. Students should have access to education on the effects of substance abuse since many students forget that substance is destructive to their health and education. Our study recommends that extensive community and state support is essential for educating students and youth. Parents, guardians, caregivers and educational authorities must cooperate and be open-minded to offering mental illness services to affected students.
